# A Novel International Partnership for Actionable Evidence on Urban Health in Latin America: LAC‐Urban Health and SALURBAL

**DOI:** 10.1002/gch2.201800013

**Published:** 2018-06-19

**Authors:** Ana V. Diez Roux, S. Claire Slesinski, Marcio Alazraqui, Waleska Teixeira Caiaffa, Patricia Frenz, Ricardo Jordán Fuchs, J. Jaime Miranda, Daniel A. Rodriguez, Olga L. Sarmiento Dueñas, José Siri, Alejandra Vives Vergara

**Affiliations:** ^1^ Urban Health Collaborative Dornsife School of Public Health Drexel University Philadelphia 19104 PA USA; ^2^ Instituto de Salud Colectiva Universidad Nacional de Lanús Buenos Aires Argentina; ^3^ School of Medicine Federal University of Minas Gerais Belo Horizonte Brazil; ^4^ School of Public Health University of Chile Santiago Chile; ^5^ Economic Commission for Latin America and the Caribbean Santiago Chile; ^6^ School of Medicine Universidad Peruana Cayetano Heredia Lima Peru; ^7^ Department of City and Regional Planning The University of California Berkeley Berkeley 94704 CA USA; ^8^ Department of Public Health University of the Andes Bogota Colombia; ^9^ International Institute for Global Health United Nations University Kuala Lumpur Malaysia; ^10^ Department of Public Health Pontificia Universidad Católica de Chile Santiago Chile

**Keywords:** health equity, interdisciplinary collaboration, Latin America, sustainability, urban health

## Abstract

This article describes the origins and characteristics of an interdisciplinary multinational collaboration aimed at promoting and disseminating actionable evidence on the drivers of health in cities in Latin America and the Caribbean: The Network for Urban Health in Latin America and the Caribbean and the Wellcome Trust funded SALURBAL (Salud Urbana en América Latina, or Urban Health in Latin America) Project. Both initiatives have the goals of supporting urban policies that promote health and health equity in cities of the region while at the same time generating generalizable knowledge for urban areas across the globe. The processes, challenges, as well as the lessons learned to date in launching and implementing these collaborations, are described. By leveraging the unique features of the Latin American region (one of the most urbanized areas of the world with some of the most innovative urban policies), the aim is to produce generalizable knowledge about the links between urbanization, health, and environments and to identify effective ways to organize, design, and govern cities to improve health, reduce health inequalities, and maximize environmental sustainability in cities all over the world.

## 1 Introduction

For the first time, more than half the world's population lives in urban areas; by 2050 this figure is expected to approach 70%.^1^ The dense intersection of the social, built, and natural environments in cities brings many benefits but also poses serious risks to both human health and the environment. Choices about how cities develop and grow, how they organize transportation and land use, how they manage access to quality housing and other resources, and the social and economic policies they prioritize and implement will have profound consequences for the health and environmental sustainability of cities. Cities are also home to large social inequities. Because of residential segregation by class and ethnicity, these social inequities are often manifested spatially as large differences across city neighborhoods in social and physical environments. Social inequities and differences across neighborhood environments have profound impacts on health. The sheer concentration of population in cities and the possibility to intervene proactively to promote the creation of healthy environments present an opportunity to cities not only to improve population health but also to reduce inequities in health and promote environmental sustainability.

The past 15 years have witnessed increasing collaborations between urban planners, public health researchers, and other social scientists in the study of how urban neighborhood and urban design may impact health and health inequities, but this research was initially largely focused on high income countries.^2^, ^3^ More recently, researchers from the highly urban or rapidly urbanizing regions of the global south have also begun to explore how city living affects health or how urban policies could be used to improve health.^4^, ^5^, ^6^ Discussions of urban health in the context of the New Urban Agenda and the Sustainable Development Goals have focused primarily on access to health care and to some extent on traditional public health issues of water and sanitation and infant mortality with limited discussion of the impact of urban environments on the new epidemics of chronic disease and injuries.^7^, ^8^, ^9^ In addition, there has been limited research into the effects of urban policies on health or health inequities.

Several features of Latin America make it uniquely suited to study how urban environments affect health and how urbanization can be managed to create a sustainable and healthy future for the planet. At 80% urban, the Latin American region is substantially more urban than other regions such as Africa (40%) and Asia (48%), although both of these regions are urbanizing rapidly.^1^, ^10^ The region now contains 20% of the largest cities in the world.^10^ Latin American cities encompass not only many megacities but also very large numbers of rapidly growing (“emerging”) small and middle‐sized cities. Latin American cities also vary in physical, economic, and social environments. This heterogeneity in size and in other features across Latin American cities can be exploited to investigate how urban environments influence health and environmental sustainability.

Another unique feature of Latin American cities is that many city governments have begun to implement policies and interventions that could impact not only health but also long‐term urban environmental sustainability (see, e.g., refs. 11, 12, 13). These include innovations in sustainable transportation (including mass transit and active transportation), urban redevelopment, food policy, and social programs, among others. By studying these policies (via case studies or “natural experiments”), including how and why they were implemented and what their health and environmental impacts might be, we can build a critical evidence base relevant to improving urban health worldwide.

Last but not least, the Latin American region is also characterized by large social inequalities. These social inequalities are manifested in cities: 19 of the world's 30 most unequal cities are located in Latin America.^14^ These social inequalities have profound implications for health and health equity. Despite a long tradition of documenting health inequalities in high income countries, there is limited documentation of how health inequities vary across cities, or of what factors or policies ate associated with smaller or larger urban health inequities by class, ethnicity, gender, or place. Understanding the drivers of these inequities, and the policies that might be most beneficial in reducing them, remains a critical need.

This article describes the origins and characteristics of an interdisciplinary multinational collaboration aimed at generating and disseminating actionable evidence on the drivers of health in cities in Latin America with the goals of supporting healthy urban policies in the region while at the same time producing generalizable knowledge for urban areas across the globe. The collaboration emerged both to address critical data, research, and policy gaps related to urban health in the region, and to take advantage of the Latin American context to generate knowledge relevant to other regions, especially those in the global south. We describe the processes, the challenges, as well as lessons learned to date in launching this novel initiative. Given the novel nature and the complexity of this multicountry partnership, we describe in detail the history of the collaborations as well as the steps the partnership has taken to formulate a common vision and agenda, and to create the basic administrative structures necessary to function in a way that is participatory, collaborative, and productive. We view the creation of both the vision and the structures as fundamental to the long‐term sustainability of the partnership and to capacity building in the region generally.

### 1.1 Origins of the Initiative: The Network for Urban Health in Latin America and the Caribbean

Building on growing interest in urban health in the Latin American region (including urban health initiatives led by the Pan‐American Health Organization and the first International Society for Urban Health conference in the region in Belo Horizonte in 2011), representatives of academic institutions and international organizations came together convened by the Dornsife School of Public Health in September of 2015. The group, which included representatives from Argentina, Brazil, Peru, and Colombia as well as the United Nations University International Institute for Global Health (UNU‐IIGH) and the Economic Commission for Latin America and the Caribbean (ECLAC), identified urban health research, capacity building, and policy translation as key needs and opportunities for the region.

To address these needs, the group agreed to constitute the Urban Health Network for Latin America and the Caribbean (LAC‐Urban Health) with three key goals: (1) to stimulate policy‐relevant research on the state and drivers of health in cities of Latin America and the Caribbean (LAC); (2) to disseminate information and findings to policy‐makers and the public; and (3) to promote translation of existing knowledge into actions that improve health and reduce health inequities, and to provide insights relevant to urban areas worldwide. The group described itself as a “regional learning network” and emphasized the critical role of exchanges and collaborations focused on research, training, and translation. The Network developed a set of eight guiding principles for its work (see **Table** **1**). These principles have proven to be important value statements that have guided the partnership as it developed.

**Table 1:** Guiding principles of the Network for Urban Health in Latin America and the Caribbean (LAC‐Urban Health)

The value of describing the state of urban health (including both physical and mental health) including comparative studies over time and across cities in the region.
The importance of understanding the particularities of urbanization (and the potential health consequences of urbanization) in the historical, economic, social, and political context of LAC.
Recognition of the multidimensional nature of health in cities, its roots in social, environmental, and other contextual determinants, and therefore the need to seek health solutions beyond the health sector.
Understanding how “places” can affect health and the role of place‐based initiatives and policies.
The critical importance of addressing large health inequities within and across cities.
Promoting capacity building and exchange of trainees.
The need to incorporate multiple methodologies including qualitative studies and historical analysis, observational studies, experiments and natural experiments, and systems modeling approaches.
The need to understand the health consequences of a range of policies currently being implemented in cities of LAC, not only from the point of view of specific evaluation of policies but also to generate knowledge on the drivers of urban health generally. This necessitates partnerships between researchers, decision‐makers, and communities, and a new way of thinking about the links between research and action.

Over time, LAC‐Urban Health has grown to over 75 members from many disciplines from more than 10 countries in the region. Regular in‐person meetings, a monthly newsletter, a website (http://www.lacurbanhealth.org/), and an active social media presence keep members informed and connected. New collaborations have already emerged. Most importantly, the Network became the context that gave rise to the Wellcome Trust‐funded Salud Urbana en América Latina (SALURBAL) project, through which the Network is working toward its foundational goals.

### 1.2 Making the Aims of the LAC‐Urban Health Network a Reality: The Wellcome Trust‐Funded SALURBAL Project

The SALURBAL proposal, funded by the Wellcome Trust in 2017 as part of the “Our Planet, Our Health” initiative was crafted by multiple members of LAC‐Urban Health as a platform through which they could achieve the network's research and policy translation priorities while remaining faithful to the network's guiding principles. The sense of cohesion, mission, and focus created by the Network were fundamental to the success of the team in developing the proposal.

The project aims and approach were developed through an extensive participatory process including an in‐person workshop and subsequent electronic communications that brought together experts in epidemiology, medicine, urban planning and transportation, nutrition, physical activity, environmental and occupational health, sociology, politics, and economics from across the region. A history of successful prior collaborations between subset of team members was critical to the success of this important phase of proposal development.

**Figure 1:**
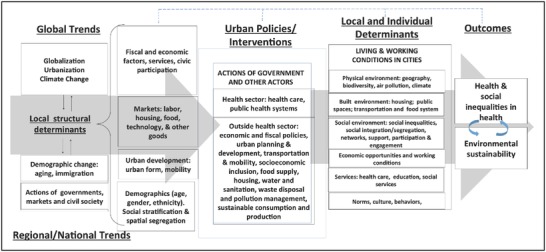
Conceptual model of key drivers of urban health, equity, and sustainability.

A key element of the proposal development phase was the creation of an agreed‐upon conceptual model of the drivers of the health and environmental sustainability of cities (**Figure** **1**). The process of developing such a model began during prior meetings of LAC‐Urban Health but it was rendered more focused by the development of the SALURBAL proposal. Key elements of the model include: (1) multilevel determinants, ranging from global trends to local neighborhood environments and individual‐level characteristics; (2) dynamic processes involving feedbacks and dependencies; (3) interrelated nature of social processes and the physical environment; (4) involvement of different stakeholders—civil society, government, nonprofits, businesses, international organizations; (5) role of policies and practices within and outside the traditional health sector; and (6) interrelated nature of human health and environmental sustainability. The model adds to previous urban health frameworks by emphasizing the critical role of urban policies/interventions and by highlighting the interrelatedness of health, equity, and environmental sustainability.^15^

Based on this conceptual model, gaps identified in the available research, and consideration of unique opportunities in the Latin American context, including a desire for comparative and innovative approaches, the team designed four overall project “aims” under which project activities are organized.

## 2 SALURBAL Aims and Approach

**Figure 2:**
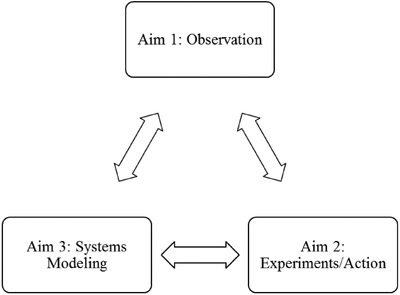
Sources of evidence for urban health knowledge and action in SALURBAL Aims.

The SALURBAL project leverages a large, multicountry interdisciplinary team spanning 16 institutions and 7 countries across Latin America (in addition to three U.S. academic partners and two United Nations entities), a multiplicity of data sources, heterogeneity within and among Latin American cities, and intersectoral partnerships to enhance understanding of how changes in urban environments affect health, social inequities in health and environmental sustainability across the globe. The project is structured around four aims: three empirical/analytical aims and one policy dissemination aim. The three empirical/analytical aims were designed to be complementary and reinforce each other over the course of the project (see **Figure** **2**).

The fourth aim is a critical dissemination and translation aim that seeks to communicate the results of the project but also more broadly to promote a new way of thinking about urban health and its drivers among policy makers and the public. SALURBAL aims and key research questions and activities are summarized in **Table** **2**.

**Table 2:** SALURBAL aims, sample research questions, and core activities

Project aim	Sample questions or objectives	Core activities
Aim 1: To quantify the contributions of city and neighborhood‐level factors to differences in levels of health and health inequalities among and within cities.	(1) What is the impact of city‐level and neighborhood‐level factors on levels of population health in cities? (2) What is the impact of city‐level and neighborhood‐level factors on the magnitude of social inequities in health within cities? (3) What city and neighborhood‐level factors are associated with beneficial health and environmental indicators?	Compile link, and document a rich harmonized data resource on health, city, and neighborhood‐level factors in the Latin American region. Units and variables are being defined at each level allowing for a variety of flexible analytical approaches, including multilevel and longitudinal analyses.
Aim 2: To evaluate the health and environmental impact of city and neighborhood‐level policies/interventions by capitalizing on natural experiments and by combining quantitative and qualitative approaches.	(1) What policies or interventions are associated with better population health and lower health inequities within cities? (2) What policies or interventions are related to beneficial urban environmental conditions and lower inequities in exposures to adverse urban environments? (3) What policies or interventions result in the most beneficial health and environmental trajectories?	The project team identified four thematic areas of interest based on interventions and policies currently prioritized in the region: mobility and emissions control, comprehensive urban development policies, reduction of social inequities, and promotion of healthy behaviors. Strategic opportunities for health impact evaluation using quantitative and qualitative approaches are being identified through working groups and a call for proposals.
Aim 3: To employ systems thinking and formal simulation models in order to (1) better understand the dynamic relations between the urban environment, health, and environmental sustainability; and (2) identify the plausible impacts of selected policies under varying conditions and dynamic relations.	(1) What are the dynamic relations between the urban environment, health, and environmental sustainability; and (2) what are the plausible impacts of selected policies under varying conditions and dynamic relations? The team identified two areas for systems modeling based on interest in the region, team expertise, and relevance of systems approaches: transportation and food policy.	Conduct facilitated workshops[16,17] engaging scientists, policy‐makers, and representatives from civil society in order to promote systems thinking and generate causal loop diagrams. This stage will help identify key systems components and refine the research questions In a second stage, we will use system dynamics or agent‐based modeling[18,19] to address key questions identified.
Aim 4: To engage with the scientific community, the public, and policy makers in order to disseminate findings and translate them into policies and interventions.	Objectives include: (1) to promote new ways of thinking about drivers of urban health and the types of policies and interventions that could improve health and sustainability in cities. (2) To engage various stakeholders in research and evaluation process in order to shape questions and facilitate dissemination. (3) To disseminate our vision and our findings broadly. (4) To advocate for and support the translation of research findings into policies and interventions.	Conduct stake holder mapping. Incorporate policy‐make input at multiple steps in the project through workshops and other events. Implement rapid research dissemination and policy translation activities across digital, print, and in‐person platforms. Evaluate impact.

**Tables** **3** and **4** provide examples of the data that will be compiled by SALURBAL and of the types of policies that will be studied for health and environmental impact.

**Table 3:** Examples of health and environmental data to be compiled by SALURBAL

Domain	Sample measures
Health	Mortality data by cause, survey data on physical and mental health and health risks factors in children and adults, objective measures and hospitalization data when available
Economic and social structure	Poverty and income, Gini coefficient, GDP, unemployment, education
Built environment	Urban footprint/land cover, compactness, density, street and intersection density, public transport infrastructure by type, active transport infrastructure, water, and sanitation
Emissions and natural environment	Air pollution, surface temperatures, green space
Social and behavioral environment	Violence, social cohesion, travel mode and motorization, housing, transit fares, gasoline cost
Organizational/Institutional factors	Governance, social services, health care, municipal taxation, land use plan, hazards plan, transit subsidies

**Table 4:** Examples of policy themes and expected impacts that could be assessed

Policy themes	Sample expected impacts
Mobility and emissions control. This includes policies related to mobility and emissions control (transit/cycling infrastructure, vehicle restrictions).	Proximal impacts on air quality, traffic, availability/density of transit/cycling infrastructureDistal impacts on physical activity levels (and travel mode), weight, respiratory conditions, cause‐specific mortality
Comprehensive urban development. This includes policies related to land access and housing subsidies and comprehensive housing/ education/ health programs in poor areas, water/sanitation.	Proximal impacts on crime, availability of sanitation and clean waterDistal impacts on child health and nutrition and growth indices, behaviors and risks factors, injuries, mortality
Social inequities. This includes policies related to social inclusion and reduction of social inequities (e.g., conditional cash transfers, minimum wage).	Proximal impacts on food security and dietary quality, nutrition status and growth indices, mental healthDistal impacts on child health and mortality, adult risk factors, violent deaths
Promotion of healthy behaviors. This includes policies related to food, leisure physical activity, and tobacco control (e.g., taxes and regulations regarding availability and labeling).	Proximal impacts on food security and nutrition; smoking prevalence, intensity, and cessationDistal impact on malnutrition (overweight and underweight), diabetes, hypertension, cancer, mortality

## 3 Organization and Coordination

A critical component of a large, multicountry and interdisciplinary initiative like SALURBAL is ensuring an organizational structure that facilitates and promotes engagement of all partners while ensuring productivity and the ability of the project to meet its aims. To address this organizational challenge from the very beginning, SALURBAL developed an organizational and governance structure inspired by other multisite projects.

The project is led by the Principal Investigator (PI) with assistance from an Executive Committee composed of five members representing different countries and project cores. The Executive Committee meets regularly to review study progress, troubleshoot any problems, and develop strategies and processes to operationalize the activities necessary to achieve study aims. A larger Steering Committee, including members of country hubs and cores (representing all institutions) meet via video conference call monthly to set broad strategies, establish priorities, monitor progress, and provide input to the PI and the Executive Committee. The entire research team meets in person twice a year to review study progress.

A Publications and Presentations Committee (P&P) reviews proposals for presentations and publications. It ensures that priority papers identified by the Executive Committee are completed; promotes the quality of presentations and publications; tracks progress; and identifies gaps and promotes publications. The Executive Committee is serving as the P&P committee during the initial phase of the project. An Ancillary Studies Review Committee will be constituted to oversee the review and selection of proposals for ancillary studies.

SALURBAL has convened science and policy advisory boards to obtain timely and expert external advice. The science advisory board provides input into the scientific priorities and approaches of the study and connects the project to other endeavors in related areas. It is integrated by scientists and researchers from a range of relevant fields. The policy advisory board provides input on project priorities with an eye toward policy relevance, provides guidance on policy translation, and connects the project to external stakeholders and policy‐makers. It is integrated by representatives from government, nonprofits, and other partners such as development banks.

**Figure 3:**
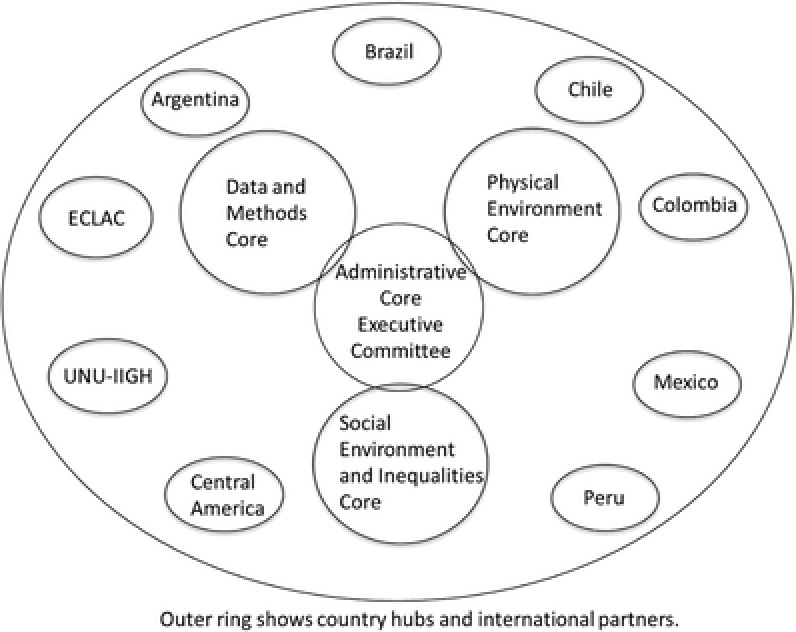
Administrative and organizational structure.

To achieve study aims the project team is organized into project cores (an administrative core plus three resource cores) and country hubs (see **Figure** **3**). The *administrative core* provides administrative oversight and coordination. It facilitates interaction and collaboration across country hubs and project cores through project meetings and video conference calls. It engages the advisory boards and supports the review or paper and ancillary studies proposals. In addition, the administrative core coordinates various dissemination, policy and community outreach activities.

*Resource cores* (data and methods, physical, and social environment cores) have the function of integrating activities and facilitating the development of collaborative products across countries and institutions involved in the project. Cores work collaboratively and with country hubs. Country investigators are affiliated with one or more cores as appropriate to their expertise and interests.

The *data and methods core* (DMC) provides overall data and analytical support to the project. The core pools health and other datasets, harmonizes variables, performs data linkages, and provides statistical and GIS expertise for all analyses. The core also provides expertise on systems approaches This core is responsible for rigorously protecting data privacy, which is especially complex due to the geographically specific nature of the data SALURBAL works with.

*The physical and built environment core* provides expertise on the measurement of the physical and built environment, on the identification and characterization of interventions or policies affecting the physical and built environment, and on relevant research questions involving the physical and built environments (or policy interventions on these environments).

*The social environment and inequities* core provides expertise on the measurement of the social environment and social inequities, on the identification and characterization of interventions or policies affecting the social environment or social inequities, and on relevant research questions involving the social environment and social inequities (including policies and interventions in this domain).

Each *country hub* is led by one lead or two colead institutions (and a country PI or Co‐PIs) that coordinate and integrate activities at all institutions engaged with the project throughout the country. Country hubs facilitate access to appropriate health, mortality, environment, and other datasets; they actively engage in data harmonization efforts, data analyses, and in the development of manuscripts for presentation and publication as lead authors or coauthors; they contribute scientific and data expertise to study cores and they engage in the dissemination of evidence to policy‐makers and communities.

In addition to the resource cores, various working groups have been organized focused on specific areas of emphasis. A systems working group has been constituted to advance Aim 3. A food environment working group is coordinating analyses related to food environments and health. Working groups focused on urban redevelopment and mass transit are exploring opportunities for health impact evaluation of urban development activities and the installation and implementation of new transit interventions.

## 4 Progress to Date

Since its launch, SALURBAL has made substantial progress. A critical component has been the consolidation of the organizational and governance structure described above, with input and participation from all country teams.

The DMC has worked collaboratively with researchers from the project's country hubs to identify two key foundational elements of the research: (1) identification of the “universe” of SALURBAL cities, including ways of operationalizing them so that they can be linked to available health data; (2) the compilation, harmonization, and geographic linkage of mortality and survey data. The DMC has also created a flexible data structure composed of cities, subcity units, and neighborhoods that can be linked to health, mortality, and other data at different levels allowing a flexible set of analyses. The physical and social environment cores have each identified a core set of domains and the levels at which they can be characterized and have initiated the process of data compilation for each domain.

The project has launched a policy scoping paper aimed at characterizing the range of potentially health relevant urban policies that are currently being implemented or have recently been implemented in cities of the region. The review will identify research gaps and useful examples for future analysis, guiding policy evaluation work during the subsequent years of the project. Simultaneously, SALURBAL is working with various potential partners to identify policies and interventions that are planned for construction or initiation during the first two years of the project's lifespan. The team plans to take advantage of these natural experiments to understand their impact on health, equity, and environmental sustainability.

Aim 3 activities to date include a training for 15 team members in the tools of participatory group model building. Following this training the team developed plans for three regional workshops with a diverse set of stakeholders (government, nonprofits, community members, international organizations, academics). Workshop goals include: (1) to engage diverse stakeholders in the SALURBAL project and vision; (2) to provide stakeholders with experience in the application of systems approaches to urban health problems, and (3) to obtain stakeholder input that will help identify and prioritize research questions to be pursued by the SALURBAL project using systems modeling in the future. The first workshop was held in Lima in November 2017. As part of the activity, stakeholders developed causal loop diagrams illustrating the relations between transport policies and health and food policies and health. Two additional workshops are planned for the first half of 2018. The results of the workshops will be summarized in a report and a manuscript for publication. To further focus systems work, the team has also launched two systematic reviews (on transport and food policy) that will inform the focus of future modeling efforts.

The project has developed a research dissemination and policy translation strategy and has launched several related activities. These include the creation of formal communication channels to LAC‐Urban Health members such as a monthly newsletter and an active social media presence. A public website for LAC‐Urban Health, which will be a primary platform for research dissemination, has been under development. SALURBAL has hosted two policy‐maker‐focused events (Bogota, May 2017; Lima, November 2017) attached to regional meetings. In these events, local policy‐makers presented on challenges and opportunities they face in improving health in cities using an intersectoral perspective. These events have resulted in one policy brief on sustainable transportation and health (already produced) and a second brief on food systems and health (under development). The project has also been active presenting work at in various venues including the International Society for Urban Health and the World Urban Forum.

To jumpstart the development of papers, the team engaged in a participatory process to identify promising “low hanging fruit” ideas that can be pursued as scientific papers early in the project. This had multiple objectives including team building, identifying early feasible but also impactful papers, and focusing the data compilation efforts. Researchers from all participating countries together with LAC‐Urban Health members present at the first in‐person SALURBAL meeting heard presentations on what data might be available during the first year of the project (including health survey data, mortality data, census data, physical and built environment data, and social environment data) and worked in small groups to brainstorm how they could capitalize on that data to answer more specific research questions during the first two years of the project. These small working group sessions were organized around five general themes: mortality data, urban form and physical environment data, social environment data, food environment data, and survey data. These discussions resulted in a preliminary list of research questions that was subsequently refined and prioritized. Writing group members were identified through a participatory process. Paper proposals based on these ideas (as well as new ideas emerging subsequently) have been reviewed by the Publications and Presentations Committee and data compilation efforts linked to these proposals are underway.

## 5 Lessons Learned

SALURBAL is still in its early stages. However, a few key lessons that may be relevant to similar initiatives across the global south have already emerged.

### 5.1 Building the Team

The team is critical to the feasibility and success of a project like SALURBAL. Several sets of team members had collaborated in various capacities before SALURBAL, but others had not. Once a core set of investigators had been identified, the team worked to expand the group via networks to encompass countries that were not represented and to expand on the expertise and disciplines included. The existence of the fledgling LAC‐Urban Health as well as the goals and values of the network were fundamental to the ability of the team to come together as it created a platform within which SALURBAL could be nested.

SALURBAL aims are perfectly aligned with LAC‐Urban Health goals, and thus SALURBAL became a project through which the network can pursue its agenda. An important aspect of the SALURBAL team is the openness to the engagement and inclusion of researchers, especially junior researchers, who are not formally supported through SALURBAL. Three key strategies have been used to promote the engagement of others throughout the region: (1) All LAC‐Urban Health members have been invited to SALURBAL meetings and selected members have been supported to attend SALURBAL meetings through additional funds provided by the network sponsors; SALURBAL meetings have also included a third day focused on Network activities; (2) the paper development and paper proposal process is open to outside investigators in order to encourage their engagement and participation; and (3) SALURBAL actively encourages and supports additional grant submissions that are synergistic and complementary with SALURBAL aims in order to facilitate the funding of investigators not currently supported through SALURBAL.

### 5.2 Organizational Structure and Processes That Promote Transparency and Engagement

SALURBAL has invested considerable effort in creating a structure that promotes strategic leadership, the development of various policies and guidelines, engagement, and accountability. The Executive and Steering Committee meetings are used to discuss and agree on complex issues such as how data will be obtained and shared, how publications will be coordinated, and to review goals and agendas for major meetings as well as to review work plans for various parts of the project. They also serve to share and socialize important information.

The creation of policies, such as the publications policy, in ways that promote and enhance collaboration with clear guiding principles in mind is also fundamental. For example, the SALURBAL Publications Policy clearly articulates four guiding principles: (1) to support and encourage the development and publication of high‐quality papers related to the aims of the project among all team members; (2) to ensure that the project fulfills its top scientific priorities across a range of areas and its deliverables to the funder; (3) to ensure that all team members have the opportunity to participate in publications; and (4) to foster capacity building for the entire team as well as the career development of junior and early stage investigators through their appropriate involvement in the publication process. The process for jump‐starting SALURBAL papers was also designed and implemented with the goals of maximizing transparency and participation while ensuring productivity and accountability.

### 5.3 Balance of Practicality and Aspirations

A key lesson that has emerged is the importance of balancing aspirations with practical realities. This implies being ambitious in goals but also being practical in the sense of identifying the “low‐hanging fruit” activities that can move forward at a faster pace while more sophisticated analyses and activities are being developed. The use of data is a case in point. In Latin America, as in many countries, health data present many challenges. There are challenges in access, in harmonization, in quality, and in completeness. And yet there is much that can be done even with imperfect data, and the insights provided by rigorous analyses of imperfect data will spur data improvements in the future. Moreover, pushing forward with imperfect data often reveals that there is more data or better data than originally anticipated. SALURBAL is already discovering a wealth of untapped data relevant to urban health, as the project works to provide, for the first time, information of the heterogeneity within and between cities in the countries of the Latin American region. SALURBAL has moved forward with the principle of balancing practicality and feasibility with perfection, in ways that maximize the informativeness of the work and promote better and better work as the project evolves.

### 5.4 Careful Consideration of Early Decisions as They May Have Long‐Term Implications

Early in the process of compiling SALURBAL data it became obvious that key early decisions related to definitions and structures could have long‐term implications, and that therefore careful consideration need to be given to these foundational decisions. A key example is methods used to define and operationalize the universe of cities that would be included in the project. For example, “cities” can be defined in various ways: based on country‐specific definitions of metropolitan areas, based on administrative boundaries linked to censuses, or based on urban footprints assessed through data‐driven efforts.^10^ Various definitions have major implications for data compilation and linkages. Another key decision is the selection of a data structure that allows the incorporation and linkage of data with varying levels of aggregation and spatial specificity and that can be shared across various cores and types of data. SALURBAL addressed these challenges through deliberative discussions and the development of processes and standards encoded in study documentation that are systematic and standardized and yet flexible enough to accommodate a variety of circumstances.

### 5.5 Capacity Building as Key and Broad

SALURBAL views capacity building as integral to all its activities. The multidisciplinary team includes and supports junior and senior researchers as well as trainees. SALURBAL provides a unique opportunity to build research capacity and regional collaboration across multiple disciplines in ten countries. The project has planned specific capacity building activities such as workshops (two training activities have already occurred). The engagement of trainees and early career investigators, critical to the long‐term success of SALURBAL, has been promoted through the attendance at meetings and through a publications process and policy that explicitly encourages and supports junior investigators. However, capacity building also extends to building experience in how to work in large multicountry teams. In this sense, the creation of the organizational structure and policies of SALURBAL is a critical capacity building activity. The team is building capacity for multisite, cross‐country work in ways that are new to many in the region. Capacity building also extends beyond the SALURBAL team to regional and country stakeholders through their engagement in data processing and data sharing activities, through the support for data linkages that will benefit local partners, and through activities like the participatory group modeling workshops. In many ways, this broad view of capacity building may be one of the most lasting legacies of the project.

### 5.6 Openness to Serendipity and Unanticipated Opportunities

A critical element in projects like SALURBAL is the need to have the flexibility to be open to new opportunities and unanticipated directions that emerge as the project develops. For this reason, these projects are better conceptualized as research networks than as strictly and very tightly predefined research studies. The opportunity to be open to new collaborations and opportunities is critical to their success. A case in point is provided by Aim 2 of SALURBAL. The specific policy evaluation opportunities were purposefully left open to maximize our ability to capitalize on the most rigorous evaluation contexts. An example that has emerged is the health impact evaluation of a new aerial tram project that will be implemented in a low‐income area in Bogotá over the next year. This could not have been anticipated when the proposal was written and the flexibility in the project has allowed us to deploy expertise and resources to capitalize on this important opportunity. This flexibility and adaptability is critical to SALURBAL's ability to produce the most informative and rigorous research.

### 5.7 Sustainability and Partnerships

**Figure 4:**
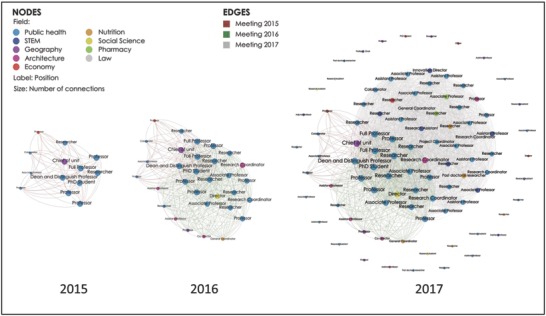
LAC‐Urban Health/SALURBAL network mapping of members and connected stakeholders in 2015, 2016, and 2017.

It is critical that projects like SALURBAL be developed with an eye toward sustainability after the funding period ends. SALURBAL has approached this by nesting the project and its communications within the broader LAC‐Urban Health Network and the Urban Health Collaborative (UHC) at the Drexel University Dornsife School of Public Health. The Network will ensure continuity of the effort after the project ends. Indeed, since initiating SALURBAL, LAC‐Urban Health has seen expansive growth of its network, which can be seen in the network map below in **Figure** **4**. We anticipate that during the five years of the project, this growth will continue, leaving behind a strong and well‐connected network primed to continue pursuing the research priorities of LAC‐Urban Health. Aligning our work with the objectives of our partners be they local academic, city governments, or international organizations is also key to creating the momentum and structures that will allow continued investment and support for this kind of work after funding for SALURBAL ends.

In addition, SALURBAL has been proactive in supporting new grant submissions even early in the history of the project to allow off shoots and supplementary activities that will expand and capitalize on the SALURBAL resource long into the future. Building partnerships and extending the project reach through continuous incorporation of new team members is also a way to ensure sustainability. SALURBAL has created partnerships with two United Nations agencies and is exploring partnerships with health organizations, development banks, and other regional stakeholders.

## 6 Challenges Ahead

SALURBAL is groundbreaking in its vision and team but it is a complex project and several challenges lie ahead. Key challenges include: (1) keeping the full team engaged despite geographic distances and heterogeneity in expertise, experience, and primary language (the team includes Spanish, Portuguese, and English speakers); (2) facilitating data access and analyses in a context that has limited experience with data sharing and pooling while protecting confidentiality and privacy; (3) producing rigorous research in a timely manner in the context of imperfect data and supporting improvements to data collection for the future; (4) capitalizing on emerging opportunities in a way that is timely and informative; (5) engaging with policy‐makers and other nonacademic stakeholders from the very beginning of the project in a way that influences the direction of the project so that its impact is truly transformative; and (6) developing strategies to communicate key facts to policy‐makers and the public in ways that are clear and impactful.

The project has aimed to position itself to address these challenges in several ways. We have a governance structure and have planned a set of systematic engagement opportunities virtually and in person that maximize engagement. We have developed a publications policy that is open and transparent and aimed at supporting and promoting the contributions of all team members. We have developed draft data use agreements that can be tailored and adapted to country needs and requirements. We have created a flexible data structure that allows us to deal with varying levels of geographic specificity and have detailed procedures for ensuring data confidentiality. Through the paper proposal development process, we have aimed to balance early productivity with more refined analyses as the project develops. Our data compilation efforts have proceeded in steps, beginning with the most easily available and proceeding to data that may be more difficult to access and process. We provide analytical and methodological support to papers in various ways, locally and centrally. Key documents are provided in English, Spanish, and Portuguese. Meetings are, at times, multilingual to accommodate all team members.

We have designed the project to allow flexibility in identifying and supporting policy evaluation opportunities as they arise. This is accomplished through funds set aside for an ancillary studies mechanism to support these activities. We have also set in place activities to engage stakeholders early in the process, not waiting until the project itself has produced new results. These activities include policy briefs based on existing knowledge, engagement in systems thinking through the group modeling workshops, and the presence of SALURBAL at events like the Ninth Word Urban Forum. We have also set in place mechanisms for evaluating the effectiveness of our policy‐maker engagement activities.

Prioritizing new collaborative opportunities and new partnerships so that the project remains open to new ideas and opportunities but still retains focus and does not get distracted from delivering on its key goals is also a major challenge. SALURBAL is addressing this challenge by exploring many new opportunities but also prioritizing our efforts and resources so that the activities we pursue are synergistic and do not distract from our key goals.

## 7 Conclusion

The mere existence of the SALURBAL project is transformative, and we have already begun to see early fruits of this ground‐breaking collaboration. There are many challenges ahead but the structures and processes we have put in place will help address these challenges as the project advances. In a transformative project of this magnitude and ambition, it is to be expected that not all will turn out as planned and that new opportunities that we did not anticipate will arise. Progress to date shows that SALURBAL is well poised to capitalize on opportunities, to overcome barriers, and to deliver on its key aims. We envision a future where other regional networks focused on urban health and sustainability may emerge, capitalizing and building on the lessons of SALURBAL, providing more relevant evidence and engagement around urban health, and partnership with SALURBAL to promote health, equity, and sustainability of cities worldwide.

## Conflict of Interest

The authors declare no conflict of interest.
